# Patient and public involvement in research published in the *British Journal of Occupational Therapy* 2015–2021: A scoping review

**DOI:** 10.1177/03080226231165374

**Published:** 2023-04-12

**Authors:** Ruth Williamson, Helen Atkin, Oliver Wood, Louise Thomson, Phillip Whitehead

**Affiliations:** 1 Leeds and York Partnership NHS Foundation Trust, Leeds, UK; 2 Northumbria University, Newcastle upon Tyne, UK; 3 NIHR Research Design Service North East and North Cumbria Consumer Panel, Newcastle upon Tyne, UK; 4 Newcastle University, Newcastle upon Tyne, UK

**Keywords:** Patient and public involvement, collaboration, multiple perspectives, GRIPP2, reporting PPI, co-authorship

## Abstract

**Introduction:**

The *British Journal of Occupational Therapy* (*BJOT*) recently introduced a requirement for authors to report Patient and Public Involvement (PPI). The public and people using health and social care services have knowledge which holds value in research beyond use as data. Involvement in research process can help shape the generation of critical evidence informing policies and practices; however, little is known about prior PPI reporting.

**Method:**

A scoping review was conducted identifying public involvement in research published in *BJOT* between 2015 and 2021. An electronic search was conducted using key terms. Data were extracted in duplicate or triplicate using GRIPP2 short form checklist.

**Results:**

Twenty-five studies were identified which reported public involvement, this was 6% of research published in *BJOT*. Patients and public were mainly involved in study steering or advisory groups and their primary activity was developing or piloting study materials. There were low levels of reporting the aim, outcome and critical reflections on involvement activities. Main approaches to involvement were consultation (37%) and collaboration (30%). There was inconsistent use of language and terms.

**Recommendations:**

Consistent PPI reporting using Guidance for Reporting Involvement of Patients and the Public (GRIPP 2), critical reflection of impact of PPI, acknowledgement of public contributors, future scoping review investigating impact of *BJOT* PPI authorship guidelines.

## Introduction

One of the key objectives for occupational therapy research in the UK is to strengthen partnerships with service users, carers, families and communities by involving them in the research process, in recognition that people with lived experience of the impact of health conditions on occupation can contribute vital knowledge and expertise (Royal College of Occupational Therapy, 2019). This approach to research, commonly termed public involvement or patient and public involvement (PPI) (NIHR, 2021a) is an established concept in health and social care research and is differentiated from working with the public as research participants; rather it is research carried out ‘with’ and ‘by’ the public, and not ‘for’ them, with public members contributing to part or all of the research process and playing a key role in shaping the outcome of the research (NIHR, 2021a). As this definition implies, an ethical mandate to rebalance power between academics or service providers and the end users of research lies behind involving public contributors as research collaborators; as such the public should be provided with opportunities to actively partake in decision-making processes that influence knowledge production.

There are multiple drivers, epistemological, moral and pragmatic behind involving the public in research. First, from an epistemological standpoint, where research incorporates the knowledge of those who have been at the receiving end of services and experienced a health condition firsthand, there is an implicit impact on research credibility and accountability unmatched by academic input alone (Glasby and Beresford, 2006; Karazivan et al., 2015). Second, public involvement can enable people to make a difference to research that ultimately impacts on their treatment options or treatment delivery, offering more control over their health; it also allows for a redistribution of power between themselves and their healthcare provider (Atkin et al., 2020; Beresford, 2013; Edelmann and Barron, 2016). For academic-led studies there is therefore a moral imperative for researchers to ‘invite the public in’, espoused by PPI national standard setters within the National Institute for Health Research (NIHR) (2019), guiding authors to adopt principles such as accountability, transparency and citizenship participation. Third, there is a pragmatic rationale behind involving public contributors in research. Authors have reported benefits such as improved study design, more pertinent and user-relevant aims, greater participant retention, improved implementation of findings (Brett et al., 2012; Domecq et al., 2014; Faulkner, 2013) and a unique opportunity for academics and lived experience experts to learn from one another (Staley and Barron, 2019).

The rationale for public involvement aligns strongly with occupational therapy’s ethos of keeping patients at the centre of practice, with critics calling for this to extend into research activity (Letts, 2003; Mroz et al., 2015; Whalley-Hammell, 2013). However, despite the profession’s intention to build strong and meaningful partnerships between public contributors and occupational therapists, questions have been raised about the extent to which the profession is really enacting the values it espouses. Karen Whalley Hammell has argued that, as a profession, occupational therapy does more to promote itself rather than those who use their services and is therefore lacking a level of self-criticality needed to ensure patient-centred values underpin all areas of the profession’s remit (Whalley-Hammell, 2007).

More recently, there are signs that public involvement is filtering through into research infrastructures in occupational therapy in the UK, such as in the Royal College of Occupational Therapy (RCOT) Research and Development strategy (RCOT, 2019) which calls for occupational therapists to involve service users in research as an integral part of credible knowledge production that can facilitate meaningful service user–academic partnerships. The RCOT also recently undertook an activity, in collaboration with the James Lind Alliance, to identify the top ten research priorities, working with five people with lived experience as part of the steering committee (Watson et al., 2021).

Recent editorials in the *British Journal of Occupational Therapy* (*BJOT*) have highlighted the importance of PPI. Harries et al. (2019) argued the need for demystification and promotion of PPI as a central research practice. Atkin et al. (2020) advocated for service users’ rights as co-researchers and called for researchers to underpin involvement approaches with values such as trust, respect and power-sharing. Significantly in 2021, *BJOT* also updated its author guidelines adding the requirement for PPI statements, stipulating authors declare what has happened in respect of public involvement in the work they seek to publish (*BJOT*, 2021; De Iongh et al., 2021). However, despite these drivers, little is known about how much public involvement has actually taken place in research published in the journal. Without a clear benchmark it will be difficult to evaluate the impact of the requirement for authors to report their public involvement activities. As such, there is a need for a better understanding of public involvement reporting in research published in *BJOT* prior to the publication of the updated author guidance. This scoping review aims to find evidence of public involvement practices in the *BJOT*. Studies use different terminology to describe public involvement. In this paper, we largely use ‘public involvement’ to encompass public, patient, consumer and service user involvement. Where other authors have used different terminology, we aimed to use their terminology when referring to their work.

## Literature review

In the UK there is a clear cultural shift to embed public involvement in research as a standard practice, reflected in the actions of those in power such as research policy-makers (NHS Health Research Authority, 2017), editorial boards of leading journals (Price et al., 2018), systematic review guidelines (Cochrane Collaboration, 2011), research funding bodies (NIHR, 2014) and the continued support of public involvement in research (NIHR, 2021b). In the literature, more nuanced changes in attitudes are reflected in terminology used to describe involvement practices. For example, terms used to describe public members have shifted from consumer and participant to terminology alluding to active role partaking, such as co-producer and co-researcher, pointing to a reduction in the hierarchy of researcher and service user and a greater inclusion of the user voice (Barnes and Cotterell, 2012; Beresford, 2013).

Despite the increased drivers for involving service users in research, problems in practising and reporting on involvement activity are prevalent. Different attitudes and approaches contribute to a debate around what ‘good’ public involvement looks like; the NIHR describes four approaches to involvement, reflecting incremental changes in user control: (1) consultation, where research is initiated by academic teams who seek public perspectives, (2) collaboration, pinning the research process around an ongoing academic–lay partnership, (3) coproduction, with collaboration between everyone involved in the research, and shared responsibility and power and (4) user-led, an emancipatory practice where decision-making lies entirely with public researchers (NIHR, 2021a). Despite one of the aims of public involvement being to redistribute power, concerns have been raised about how successfully public contributors can bring about real change through some of these approaches. User-led groups have argued that research led and controlled by users is the ‘gold standard’, with traditional academic researcher-led approaches bringing the risk of ineffective or tokenistic involvement (Survivor Researcher Network, 2018; Turner and Beresford, 2005). However, the NIHR (2019) published co-produced *National Standards for Public Involvement* which argued that principles and values, such as the need for openness and transparency between research partners, are more important than the type of approach (NIHR, 2019).

Previous studies have sought to explore the scope of public involvement in occupational therapy health research (Røssvoll et al., 2022); in the *Australian Occupational Therapy Journal* (Cox et al., 2021) and more widely (Garfield et al., 2016; Price et al., 2018; Shen et al., 2017; Smith et al., 2008), and an issue raised by these authors is that limited and inconsistent reporting of public involvement practices is widespread. It is not clear whether this is due to low levels of involvement or low levels of reporting. Notwithstanding, studies exploring academic attitudes to involving service users suggest possible scepticism towards skills public members can bring, reluctance to relinquish power and a lack of support from research funders to involve public members as research partners (Boaz et al., 2014; Snape et al., 2014).

Steps have been taken by research gatekeepers to reduce the risk of low-quality involvement and promote the values espoused by the national standard setters. A literature review by Price and colleagues reported a tenfold increase in the amount of PPI reported in research following policy implementation in the British Medical Journal (BMJ) (Price et al., 2018). Critics have advocated for more journals to follow suit, requesting authors provide adequate descriptive information about the processes involved when working with public researchers (Price et al., 2018; Staniszewska et al., 2017). Others have gone a step further by producing a critical commentary written by public researchers reflecting on the research process (e.g. Di Lorito et al., 2020; Crabtree et al., 2016). The extent to which such practices have been incorporated into research published in the *BJOT* is unclear.

The overall aim of this study was to examine public involvement reported in occupational therapy research published in the *BJOT* between February 2015 and July 2021, in order to provide a benchmark for reporting prior to the publication of the updated *BJOT* author guidance.

The specific objectives for the study were to:

1. Establish how many research papers published in *BJOT* in the past 5 years reported involving the public in the research process.
2. Explore reported ways in which the public has been involved in the research process, and their involvement in specific research activities.
3. Evaluate the reported approach to involvement taken by *BJOT* authors to understand the quality of the reporting of PPI in research and, where possible, the outcomes and the quality of the PPI activity undertaken.

### The research team for this study

Our research team comprises an occupational therapist in clinical practice, two lived experience researchers and two academic occupational therapists. We worked collaboratively throughout the research process, aiming to ensure the objectives, selection process and interpretation of findings for this review were in keeping with the needs of both end users of research and people who might adopt a lived experience researcher role in future work. We aimed to use multiple perspectives from practice, academia and lived experience to contest ideas and assumptions, using the collective experience to create new meanings.

## Method

### Definitions used and search strategy

The conduct of this review was informed by guidance on systematic scoping reviews from the Joanna Briggs Institute (Peters et al., 2020) and guidance on systematic review published by the Centre for Reviews and Dissemination (2008). The NIHR definition of public involvement in research was used to inform the search and selection processes (NIHR, 2021a). The search was designed to include strings related to the population, that is the people who were involved in the research, and the methodology used for the involvement. The population string included common terms in literature used to describe public representatives such as ‘*public involvement*’ (NIHR, 2021a). The method string included terms for concepts underpinning PPI such as ‘*collaboration*’, and ‘*consultation*’, and participatory approaches such as ‘*participatory*’ and ‘*co-production*’ (Islam and Small, 2020). The full search strategy is available in Supplemental Appendix 1.

The search was conducted in *BJOT’*s online database via *SAGE Journals* on 1st March 2020 and updated on 27th July 2021. Studies were included where:

- There was a reference to public contributors being involved in the research process
- They were classified as ‘Research’ by *BJOT*
- They were published between 1st February 2015 and 27th July 2021.

The selection process was designed collaboratively by the research team influencing critical decisions including: choosing to exclude studies where collaborators were not public contributors but were key research stakeholders, such as occupational therapy students; and choosing to include studies reporting on the co-design of interventions, using a rationale that the values of co-design align public involvement. We decided to include studies reporting involvement of public members without clear description of their activities; we deemed this an issue of minimal or poor reporting worthy of evaluation.

### Study selection

To improve the rigour of the search, all identified studies were screened in full text by the first author. The decision to read the full text at an early stage was made following a hand search in the planning stages, indicating that authors do not always clearly report on the involvement of service users in the title, abstract and keywords alone (see e.g. Craig et al. 2017; Chung, 2019). A random sample of 30% of identified studies were screened in duplicate or triplicate by the co-authors. There was a 92% level of agreement in the decisions to include/exclude and therefore further duplicate screening was not undertaken.

### Data extraction and analysis

Study-specific information was extracted which included: who was involved in the research, study type, practice area and funding body. For data extraction in relation to public involvement, the National Standards were used to guide the appraisal of involvement approaches used by authors (NIHR, 2019). Firstly, we extracted data relating to basic study information: country, practice setting, funder, methodology and who was involved in terms of public members. Secondly, the GRIPP2 short-form checklist, the GRIPP2 an evidence-based tool developed by community consensus to appraise the reporting of PPI in publications (Stanizewska et al., 2017), was adapted to include approaches to PPI, acknowledgement of the public, searchability of PPI and co-authorship. Categories of data extracted were: the aim of involving public contributors in the research; the methods used for the public involvement, which we equated to how they were involved; the results, which we equated to activities they were involved in; the outcomes of the public involvement in the study; and any critical comments on the public involvement. All data were extracted in duplicate or triplicate: initially by the first author, then by one or two other authors. Extracted data were checked for consistency and any discrepancies were resolved either by the extracting authors or by consensus amongst authors.

From the data extracted using GRIPP2, information on the method of involvement (how the public were involved) and results (what activities they were involved in) were grouped into categories and summarised in text and tables. For the remaining data, a narrative synthesis was used to explore relationships in the data, as recommended by review protocol guidelines when seeking to understand data that is insufficiently similar (Popay et al., 2006; Centre for Reviews and Dissemination, 2008).

## Findings

**Figure 1.**
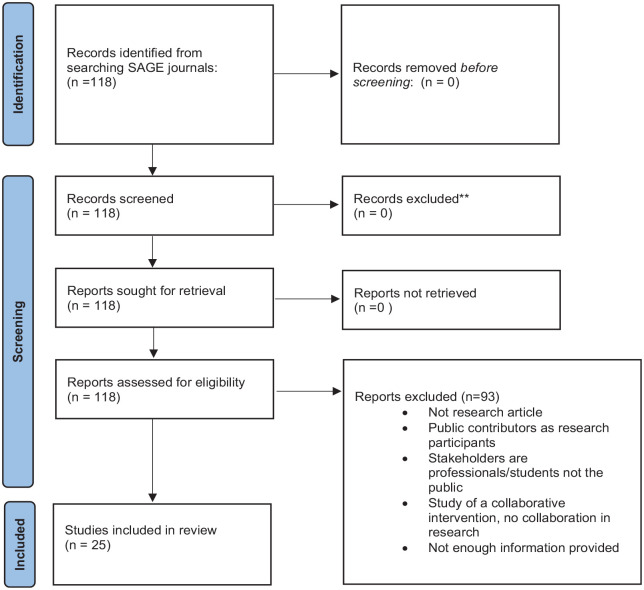
Search Process.

In total, there were 402^1^ research papers published in the *BJOT* in the print version of the journal between February 2015 and July 2021, inclusive. Applying our search terms identified 118 which were screened for inclusion in this review. Ninety-three were excluded, the main reasons being that the public were involved as participants but not in the research process, or that they were not research (e.g. practice analysis). The remaining 25 thus represented 6% of research articles published in the journal during the review period. The search process is presented in Figure 1.

The included studies are summarised in Table 1. It appears that except for 2019 with six articles published, numbers have remained broadly static at three to four since 2016. Regarding country of origin, 21 studies were from the UK, two from Australia and one from Ireland. In terms of the primary practice setting, 10 were from mental health settings, four from stroke, two from paediatrics and one each from vocational rehabilitation, homelessness, forensics, orthopaedics, neurology, palliative care and social care. The remaining study was across the profession as a whole. Seven studies were funded by the NIHR, five by the Royal College of Occupational Therapists including the United Kingdom Occupational Therapy Research Foundation, two by UK Higher Education Institutions, four did not report any funding and the remaining six each reported a different funder. In terms of study methodology, 13 were primarily qualitative, and four were mixed method with the remainder reporting different approaches as shown in Table 1. In terms of who was involved, the amount of information provided varied. Where numbers were available there was a range of one to eight service user or public members reported.

**Table 1.** Included studies.

Reference (Year)	Country	Practice setting	Who was involved?	Study type	Funder
Arblaster et al. (2019)	Australia	Mental Health	Lived experience researcher co-author; consumer reference group with lived experience	Qualitative; in-depth interviews	None
Birken and Bryant (2019)	UK	Mental Health	Service user researcher; service users on advisory panel	Qualitative: photovoice; participatory research approach	UKOTRF; Institute of Social Psychiatry Scholarship Grant
Boniface et al. (2015)	UK	Mental Health	Eight mental health service users	Qualitative: Collaborative Action Research	UKOTRF; Institute of Social Psychiatry Scholarship Grant
Bryant et al. (2016)	UK	Mental Health	Mental health service users on steering group (possibly different people at different times)	Qualitative; interview and focus groups.	None
Cameron et al. (2016)	UK	Mental Health and Work	Three mental health services users currently in work and a community group	Comparative case study and collaborative design.	University of Brighton
Chung (2019)	UK	Mental Health	Steering group with one lay member	Qualitative; interviews.	Elizabeth Casson Trust
Cook et al. (2016)	UK	Mental Health	Two service user consultants and members of the North London Service User Forum	Consultation events; intervention piloting	NIHR
Coole et al. (2020)	UK	Vocational Rehabilitation	Two service user representatives on steering group	Mixed methods; intervention design and feasibility study.	UKOTRF
Craig et al. (2017)	UK	Mental Health/Workplace	Lived experience experts from the ‘Imagine Programme’	Qualitative; diary writing and thematic analysis.	ESRC
Drummond et al. (2020)	UK	Stroke	Public and Patient Involvement Group (not clear how many)	Mixed methods; survey, interviews, focus groups. Intervention co-design.	UK Stroke Association
Farr et al. (2017)	UK	Paediatrics	A parent consultant as co-author; parent consultation group.	Mixed methods; questionnaire yielding qualitative and quantitative data.	NIHR
Fieldhouse and Greatorex (2020)		Homeless	One former service user on the steering group	Qualitative; interviews and focus groups.	1,625 Independent People
Honey et al. (2019)	Australia	Mental Health	Three lived experience researchers with research experience were co-authors	Qualitative; collaborative auto ethnography	Sydney North Shore and Beaches Partners
Jones et al. (2022)	UK	Physical Health	Three pre-established PPI groups from health and social care utilised as ‘brainstorming groups’.	Qualitative: participatory approach using co-design workshops	Health and Social Care Partnership Sheffield Health and Care Challenges Collaboration Forum
Jones and Nasr (2018)	UK	Stroke	A patient and public panel involved in co-design of the study	Qualitative; focus groups and photo elicitation.	NIHR
Khalifa et al. (2020)	UK	Forensic/Workplace	Lived experience Advisory Panel	Qualitative; interviews.	NIHR
Lightfoot et al. (2018)	UK	Orthopaedics	One patient representative on the research team	Trial protocol	University of Nottingham
Lovegrove et al. (2017)	UK	Neurology	People with Parkinson’s disease involved in a patient and public consultation	Qualitative; interviews and thematic analysis.	None
Lynch et al. (2020)	Ireland	Paediatrics	Research advisory group included parents and other stakeholders	Qualitative; multi-method case study,	Centre for Excellence in Universal Design
Phillips et al. (2019)	UK	Stroke	PPI representatives – no other information	RCT – extended follow up	UKOTRF
Talbot et al. (2018)	UK	Mental Health	Expert by experience advisory panel. The project steering group also had members from a service user reference group	Implementation study	NIHR
Taylor and Jones (2017)	UK	Palliative Care	People with experience of living with cancer co-designed a tool	Participatory approach	None
Elizabeth Tremayne et al. (2021)	UK	Stroke	Three people with lived experience	Qualitative; semi structured interviews.	Health Education England/NIHR
Watson et al. (2021)	UK	All	Five people with lived experience	Consensus study	RCOT
Whitehead et al. (2018)	UK	Social Care	Lay members were included in the project steering group	Mixed methods	NIHR

Overall, there was a lack of consistency in authors’ reporting of public involvement. Some studies did not provide adequate descriptive detail about approaches authors used when working with the public as research partners, making it difficult to assess the quality of involvement in some cases. This particularly applied to the following areas from the GRIPP2 short form: the aim of the PPI, the discussion of the PPI and critical reflections on the PPI.

**Figure 2.**
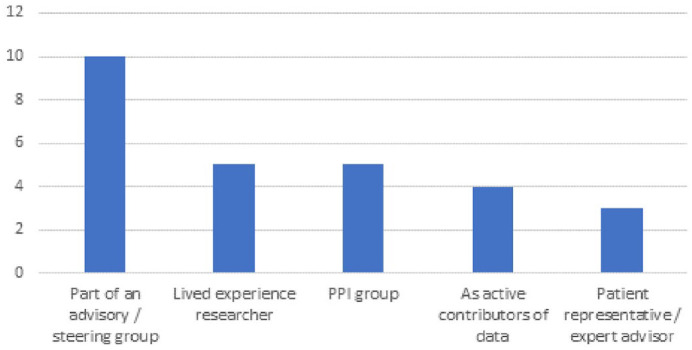
How public members were involved?

Figure 2 shows how public members were involved in the included studies. The most common method, in ten (40%) studies, was as members of a steering group which usually also included other stakeholders. This was followed by involvement as research team members (lived experience researchers) (*n* = 5, 20%), as part of a separate PPI group in (*n* = 5, 20%) and as ‘patient representatives’ or ‘expert advisors’ (*n* = 3, 12%) where the exact mechanism for their involvement was not specified. In four studies (16%) public members were involved as research participants and, although this was an exclusion criterion the authors described these activities using terms associated with PPI. Including articles which involved public members as research participants led to considerable discussion and debate. In these four studies, public contributors were involved in contributing data to either shape a research agenda or co-design a practice initiative. Whilst researchers did not always involve the public in the design of the specific research project, they did articulate that they involved the public in shaping the research agenda or practice initiative. We discuss the challenges of categorising data further in the discussion section. The total number of ways the public were involved is greater than the total number of included studies as in Arblaster et al. (2019) and Farr et al. (2017) where the public were involved in more than one way.

**Figure 3.**
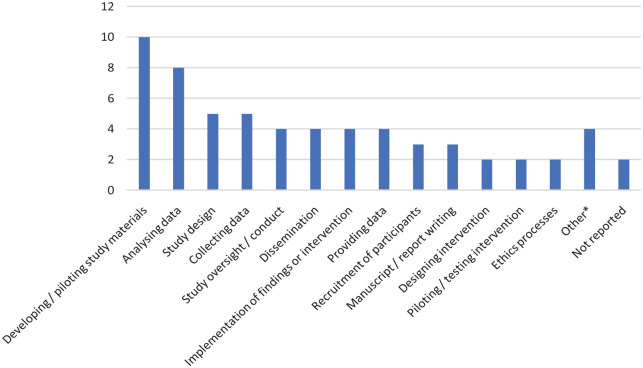
What were public members involved in?

Figure 3 shows the research activities that the public were involved in. Overall, there was a wide variety and we identified seventeen different involvement activities. In most studies the public were reported to be involved in more than one activity. The most common activity was developing or piloting study materials (*n* = 10, 40%), followed by analysing data (*n* = 8, 32%), collecting data (*n* = 5, 20%) and study design (*n* = 5, 20%). Two studies did not specifically report the activities that the public were involved in; in these studies, the public were part of an advisory group with other stakeholders and it might be assumed that this was a study oversight role.

The outcome of public involvement was discussed in 11 (44%) studies. In most qualitative studies, public input had influenced data collection and analysis processes, which research teams reported had improved the trustworthiness of data collected. Benefits for the research participants were reported in three studies (12%) with service user involvement helping to ensure the process was less burdensome for them and making interviews more inclusive (Jones and Nasr, 2018), and fostering stronger and more trustworthy relationships between researchers and participants (Arblaster et al., 2019; Bryant et al., 2016). For those undertaking the research, benefits were reported in one study (Boniface et al., 2015), where service users were said to feel empowered in designing an outcome measure for a mental health service. Researcher-reported benefits were improved researcher reflexivity and positively challenging academic assumptions (Arblaster et al., 2019) and gaining a deeper understanding of public contributor perspectives (Honey et al., 2019; Watson et al., 2021).

Principles of inclusivity were evident in the steps taken to address power dynamics between academics and public contributors, with nine studies (36%) explicitly stating this an intended strategy for effective involvement, through co-authorship (Arblaster et al., 2019; Craig et al., 2017; Farr et al., 2017; Honey et al., 2019) or the use of participatory action research or co-design methods (Birken and Bryant, 2019; Boniface et al., 2015; Honey et al., 2019; Taylor and Jones, 2017; Watson et al., 2021).

Strategies for improving accessibility for public contributors addressed the practical challenges of getting involved in research, including reimbursing travel costs and organising meetings in a central location (Taylor and Jones, 2017) and offering a variety of methods for participation such as meetings held online (Lovegrove et al., 2017). Accessibility was improved by providing plain language summaries of the research plan (Boniface et al., 2015) and arranging separate meetings for public and non-public researchers (Taylor and Jones, 2017).

Researcher training was reported in three studies (Birken and Bryant, 2019; Khalifa et al., 2020; Taylor and Jones, 2017) and in three papers there was a designated PPI lead (Khalifa et al., 2020; Lovegrove et al., 2017; Talbot et al., 2018). Public contributors were reported to have been paid for taking part in training in three papers (12%) (Birken and Bryant, 2019; Bryant et al., 2016; Honey et al., 2019). Lovegrove et al. (2017) raised the risk of the professionalisation of public contributors through research training, impacting on them no longer being ‘lay people’, potentially challenging the primary reason for their involvement.

Eight studies offered critical reflections on engagement with public contributors. Three expressed concerns about adequate public representation and diversity with one committing to more diverse representation in future projects (Watson et al., 2021). Another concern was that lack of information technology access impacted on involvement (Lovegrove et al., 2017; Watson et al., 2021). The pragmatics of involving public contributors were reported as the need for facilitator flexibility when collaborating with service users (Bryant et al., 2016). Strong relationships and a commitment to working in collaboration from an early stage were viewed as fundamental to PPI (Birken and Bryant, 2019; Bryant et al., 2016; Cook et al., 2016; Honey et al., 2019). Honey et al. (2019) provided extensive critical reflection about collaborative working, concluding that fostering a good relationship between academics and lay researchers was fundamental and recommending that other authors prioritise this. Reflections on the impact of PPI on services and organisations were noted particularly by Bryant et al. (2016) and Watson et al. (2021), with Watson identifying a commitment to systemic organisational change as a consequence of public contributor involvement.

**Figure 4.**
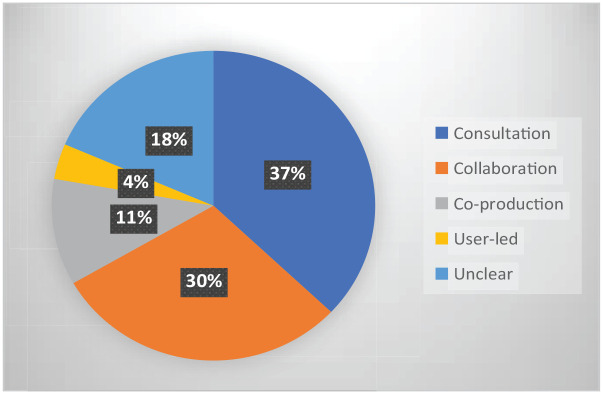
Approach to involvement.

We categorised each study into the approaches outlined by NIHR (2021a), as shown in Figure 4. Over one-third (37%) were categorised as consultation, 30% as collaboration, 11% as coproduction and one study as user-led. For five studies the approach was unclear making categorisation impossible. Two studies were categorised as both consultation and collaboration. Public members were acknowledged in 14 (56%) of the articles and were co-authors of three (12%). In four studies they were not acknowledged and in five the role of those acknowledged by name was unclear. One study had both a public member as a co-author and a clear acknowledgement of public members.

## Discussion

Overall, this review found that most of the research published in the *BJOT* between 2015 and 2021 did not report public involvement. Furthermore, most of those studies which did report public involvement contained elements of reporting which were unclear or ambiguous. The public were mainly involved as steering group members and in developing or piloting study materials. These findings represent what was reported by study authors and, as there was no previous requirement for *BJOT* authors to describe public involvement in the manuscript, the evidence is dependent on authors’ decisions to prioritise this. These findings on low and inconsistent reporting are comparable with a review of the *Australian Occupational Therapy Journal* (Cox et al., 2021) and in occupational therapy health research (Røssvoll et al., 2022) which suggest that there are low levels of reporting of PPI in occupational therapy research internationally. Funders have a key role to play in promoting public involvement in research. The majority of studies included in this review were funded by the UK National Institute for Health Research or the UK Occupational Therapy Research Foundation which both had clear mandates for PPI in research applications prior to the period covered by this review.

In order to extract information, we used a shortened version of the GRIPP2. For all studies we were able to identify the method of the involvement (e.g. as part of a steering group or separate PPI group). Twenty-three (92%) explicitly stated what the public members did, which we categorised as the results of the PPI, and the two that did not state that they were part of advisory or steering groups, which might imply they were involved in study oversight. Across the studies, we found inconsistent or poor reporting in the explicit aim of the PPI, there was little apparent consideration by most authors about the intended outcome of PPI, and little critical analysis of what went well after the study was carried out. This suggests that most authors do not use a framework, such as GRIPP2, when reporting PPI.

There were some exceptions with a higher level of reporting across all GRIPP2 short-form criteria. These tended to be studies where public contributors were key players during the whole process, usually undertaking participatory research methods (Birken and Bryant, 2019; Boniface et al., 2015; Bryant et al., 2016; Honey et al., 2019; Taylor and Jones, 2017). Here the underpinning emancipatory intentions seemed to influence increased levels of reporting. However, it is important to acknowledge other research approaches do not necessarily see public contributors as being involved so heavily and therefore lower levels of reporting would be appropriate. The NIHR (2021c) suggests that approaches will vary depending on resources and research goals, and best practice is for the public members to choose for themselves how they are involved and what they are involved in.

There were some challenges in extracting and categorising the information and we reflect on these. We conceptualised public involvement as research carried out ‘with’ the public, meaning that they were in some way involved in the research process as opposed to contributing data as research participants. In practice, this distinction is not always clear cut. This is particularly the case for participatory designs, such as Participatory Action Research which involves participants being the researchers on issues that concern them. We also found other studies where public involvement and contribution of data as a participant were conflated, for example where authors described an activity as PPI that appeared to involve data collection only. In these cases, we found ourselves considering the intention behind the public involvement in order to determine whether the intention of involvement was to shape aspects of the research processes rather than to answer the research question by contributing data only. However, this also becomes complicated where a research question is about identifying and shaping research priorities, where people who are recruited specifically to contribute the public perspective to the research process but may also contribute data.

Categorising involvement into ‘consultation’, ‘collaboration’, etc. was not a straightforward process either. Some studies were not necessarily one thing or another. For example, aspects of the study may have involved collaboration whilst others involved consultation. It seems reasonable that involvement in studies may change over time, researchers’ intentions and public contributions or commitments also. Therefore, the position may be much more nuanced; we see a value in such fluidity in these relationships and the need to move away from categorising involvement in purely static terms. We also identified some discrepancies between the terms authors used to describe their work and our interpretations and classifications. Inconsistency in the use of language and terms was a key issue in the studies included in this review and this requires further critical reflection on the part of researchers and increased clarity when reporting involvement activities.

Although reporting-related issues may distort the picture of public involvement activity, it is reasonable to infer that involvement carried out in collaboration with public contributors remains low in research published in *BJOT.* It is therefore important to highlight potential challenges research teams face in improving public involvement going forwards. Di Bona et al. (2017) relate the problem of occupational therapists’ low engagement in research practice to issues such as time constraints, confidence and managerial support. It is likely that this also impacts on public involvement. Furthermore, Oliver et al. (2019) critique co-production from the perspective of academics, describing operating within a ‘culture of hit-and-run research (get funding, do research, achieve impact, leave)’ (pg. 6). An ongoing time commitment needed to see a project through may be a real challenge, and commitment is dependent on factors such as changing health status and whether public contributors can be paid for their work.

There is a limited amount of previous similar work with which to compare our findings. The review by Price et al. (2018) compared the frequency of reporting in the BMJ in the year before and after the journal introduced a policy requiring authors to report PPI. In the year before the policy was introduced (June 2013–May 2014) 1 out of 189 research papers (0.05%) reported PPI. This was lower than the rate we found in *BJOT* (25/402, 6%). In those that did report PPI, we also found a slightly higher rate of acknowledgement of public involvement at 56% compared to 35% for Price et al. (2018) and the rates of co-authorship were the same at 12%. This suggests that the frequency of reporting of PPI in *BJOT* was higher than in the BMJ, prior to the introduction of a mandatory requirement to report. Price et al. (2018) noted an increase to 11% of research articles reporting PPI in the year following the introduction of the requirement for authors to include this; further analysis of PPI reporting in *BJOT* will be required. The review by Cox et al. (2021) reported that 48 out of 123 eligible papers (39%) in the *Australian Occupational Therapy Journal* included ‘consumer engagement’. Whilst it may appear that this represents a higher level of involvement compared to the figures reported in this review, they appear to have adopted a broader definition of public involvement than we have. For example, studies were included where participants undertook member checking of qualitative research; we would have classified this activity as research participation and not public involvement and thus excluded such studies from our review. This makes drawing direct comparisons problematic.

Based on this scoping review we specifically recommend the following:

1. For studies which are reporting public involvement, authors should aim to report a minimum of the five areas of the GRIPP2 short form (Stanizewska et al., 2017). This should include the aim, method, results, outcome and critical reflections on the PPI. In practice, we found significant overlap between these categories and suggest that additional critical reflection on the definition and application of these categories will be required by researchers when planning and reporting their involvement. There may be scope to do this through online supporting or supplementary material (as advocated by Cox et al., 2021).
2. Researchers should critically reflect on how public contributors have impacted on the production of knowledge. They should self-identify which of the four approaches (1. Consultation, 2. Collaboration, 3. Co-production or 4. User-led) they used. However, researchers should not be wedded to one static definition and should report how and why aspects of their study may have used different approaches or where approaches varied over time.
3. Service user or public contributors should receive a minimum of an acknowledgement. They should be asked whether they would like to be acknowledged by name or remain anonymous and be acknowledged as a ‘public contributor’. Co-authorship should be offered where the authorship criterion has been satisfied and additional support should be provided, where necessary, to facilitate this.

### Strengths and limitations of this study

This study involved both consultation and collaboration with people who have experience of using services and occupational therapists in practice and academic roles. We collaborated at all stages of the research process including designing the study, planning the searches, extracting data, analysing and synthesising data, and writing up study findings; our multiple perspectives enabled us to debate the nuances and complex issues encountered during the review and this is a particular strength of our research. Furthermore, we believe that this is the first review to have specifically considered the issue of public involvement reporting in this journal. Given the challenges in how public involvement is described within studies, including the range of approaches and varied terminology used by authors, there is a risk that this review may have missed some instances of public involvement during the search process. However, this was mitigated by duplicate or triplicate screening for 30% of studies with over 90% agreement between authors on decisions to include or exclude. Finally, as many studies reported minimal details about involvement, we sometimes had to use our interpretation to categorise and classify involvement, sometimes classifying as unclear, and it is possible that the researchers would have classified their studies differently. It is also possible that low levels of reporting means that the frequency and extent of public involvement in occupational therapy research may be underestimated in our findings. This warrants further investigation.

The co-authors undertaking this study found some of the categories on the GRIPP2 short form ambiguous but have included the reporting of these details in relation to this scoping review study in Table 2 below. We hope this will serve as a useful example.

**Table 2.** Reporting patient and public involvement in this study.

Area	Our comments and reflections
The aim of the PPI in the study	To share different perspectives and work together as equal partners to create new knowledge. To create new understanding by engaging in critical discussion together. Personal aims were to honour the work of all those recruited as PPI members in studies, especially those whose participation has not been reported adequately.
Provide a clear description of the methods used for the PPI (how and what)	The first phase of this project took place as a master’s study by the first author. In this stage the involvement was consultancy by both the lived experience and academic researchers. After completion of the master’s project, we agreed on a shared objective to pursue publication, and we agreed to work together as co-researchers sharing equal responsibility for all aspects of the study.
Results of the PPI in the study including both positive and negative outcomes	We agreed on the overall aims and selection criteria collectively. We each reviewed a share of all included studies independently using GRIPP2. The results of these were collated and discussed and all decisions and interpretations were agreed upon collaboratively. We participated in online discussions of overall findings, and all contributed crucially to authoring the manuscript.
Outcomes – the extent to which the PPI influenced the study overall	The multiple perspectives at all stages of the research process added value. We were able to contest ideas and assumptions and use our collective experiences to create new perspectives. We were able to assume different roles within the process – different perspectives were especially useful to understand where we had made interpretations or where there were misalignments in part of the process.
Critical comments on the PPI, what went well and what did not go well	*What went well* We shared a sense of purpose. We shared mutual respect for each other and each other’s perspectives regardless of role. We all felt that we learned from working together as co-researchers. All researchers had reservations about the ease of use of the GRIPP2, it was heartening that we all felt this. There is greater commitment to further PPI in the future and we would like to work together further as a collaborative group. Learning, feeling valued and encouraged by other researchers about value of PPI in research. There was a sense that progress will be made in encouraging reporting of PPI and in turn promoting its use. What did not go well. PPI did slow the process down as questions were asked and assumptions were challenged (see above); however, we feel the work is better for this! It took time to organise the meetings, getting everyone together. Working remotely during COVID was a challenge and our preference would be to meet face-to-face. There were IT challenges for one group member. There were difficulties in accessing funding for the PPI in this project, given the origins as a student project. It took time for the working relationship to evolve (we knew each other to different levels before beginning the work) and it took time to understand and engage the diverse strengths of the whole team. The effect of PPI on health needs to be considered as this can be positive or challenging dependent upon personal circumstances.
Approach to Involvement (e.g. Consultation, Collaboration)	As stated above, this began as a consultation due to it being a student project. However, it progressed as a collaboration with aspects of co-production.
Acknowledgement or Co-authorship	Co-authorship.

## Conclusion

This review found that most research published in *BJOT* did not report public involvement. The majority of studies that did report involvement contained minimal details or unclear components, although there were exceptions. This inhibits readers’ understanding of how public contributors impacted on the research and, importantly how public researchers impacted on knowledge constructed during the research process. There was variation in the use and application of language and key terms, which also affected understanding of involvement and comparing included studies. We have made practical recommendations which aim to improve the quantity, quality, clarity and consistency of reporting for future research published in *BJOT*.

**Key findings**

- Levels of reporting public involvement are low, indicating varying perceptions of its value
- Policy drivers to increase involvement are not increasing its visibility, which may require specific action by journal editors in submission guidelines.

**What the study has added**

Using a systematic method, this study has found low levels of reporting on public involvement in research published in *BJOT* prior to the introduction of the author requirements to report this.

## Supplemental Material

sj-doc-1-bjo-10.1177_03080226231165374 – Supplemental material for Patient and public involvement in research published in the *British Journal of Occupational Therapy* 2015–2021: A scoping review Supplemental material, sj-doc-1-bjo-10.1177_03080226231165374 for Patient and public involvement in research published in the *British Journal of Occupational Therapy* 2015–2021: A scoping review by Ruth Williamson, Helen Atkin, Oliver Wood, Louise Thomson and Phillip Whitehead in British Journal of Occupational Therapy

## Notes

1. NB – our search also included those which were published online ahead of print during this period and the figure will be slightly higher than the total published in the print journal.
