# Supplementary material for: Patient and public involvement in research published in the British Journal of Occupational Therapy 2015–2021: A scoping review
Source: Br J Occup Ther. 2023 Apr 12;86(6):400–12. doi: 10.1177/03080226231165374 (PMC12033514; doi:10.1177/03080226231165374)
Supplement: sj-doc-1-bjo-10.1177_03080226231165374 – Supplemental material for Patient and public involvement in research published in the British Journal of Occupational Therapy 2015–2021: A scoping review [file sj-doc-1-bjo-10.1177_03080226231165374.doc]

**Appendix 1 Search strategy**

|  | Search number | Search terms |
| --- | --- | --- |
| Population | **1**  TITLE & ABSTRACT | consumer OR “lay” OR stakeholder OR “end user” |
|  | **1a**  ANYWHERE | “PPI” OR “public involvement” OR “user involvement” OR “expert* by experience” OR “expert service user*” OR “expert adviser” OR “community partner” OR “patient representative” OR “user representative” OR “user reference group” |
| Method | **2**  TITLE & ABSTRACT | collaborat* OR “action research” OR “emancipatory” OR “contributor” OR “participatory” OR inclusiv* OR “consulted” OR “consultation” |
|  | **2a**  ANYWHERE | “patient voice” OR “user voice” OR “advisory group” OR “advisory committee" OR “steering committee" OR “steering group” OR coproduc* OR “co produc*” OR “co-constructed” OR “co-created” OR “user control*” |
|  | **3** | 1 OR 1a AND 2 OR 2a |
